# The Role of Autophagy in Human Uveal Melanoma and the Development of Potential Disease Biomarkers and Novel Therapeutic Paradigms

**DOI:** 10.3390/biomedicines12020462

**Published:** 2024-02-19

**Authors:** Janney Z. Wang, Paus Paulus, Yihe Niu, Ling Zhu, Christophe Morisseau, Tristan Rawling, Michael Murray, Bruce D. Hammock, Fanfan Zhou

**Affiliations:** 1 Molecular Drug Development Group, Sydney Pharmacy School, Faculty of Medicine and Health, The University of Sydney, Sydney, NSW 2006, Australia; 2 Save Sight Institute, The University of Sydney, Sydney, NSW 2006, Australia; 3 Department of Entomology and Nematology, UCD Comprehensive Cancer Center, University of California, Davis, CA 95616, USAbdhammock@ucdavis.edu (B.D.H.); 4 School of Mathematical and Physical Sciences, Faculty of Science, University of Technology Sydney, Ultimo, NSW 2007, Australia; tristan.rawling@uts.edu.au

**Keywords:** autophagy, uveal melanoma, biomarker, drug development

## Abstract

Autophagy is a form of programmed cell degradation that enables the maintenance of homeostasis in response to extracellular stress stimuli. Autophagy is primarily activated by starvation and mediates the degradation, removal, or recycling of cell cytoplasm, organelles, and intracellular components in eukaryotic cells. Autophagy is also involved in the pathogenesis of human diseases, including several cancers. Human uveal melanoma (UM) is the primary intraocular malignancy in adults and has an extremely poor prognosis; at present there are no effective therapies. Several studies have suggested that autophagy is important in UM. By understanding the mechanisms of activation of autophagy in UM it may be possible to develop biomarkers to provide more definitive disease prognoses and to identify potential drug targets for the development of new therapeutic strategies. This article reviews the current information regarding autophagy in UM that could facilitate biomarker and drug development.

## 1. Introduction

Cellular homeostasis is controlled in part by the balance between protein synthesis and degradation. The regulation of protein turnover is achieved through mechanisms that sense environmental changes to activate protein synthesis or degradation [1]. Autophagy regulates the degradation, removal and recycling of intracellular components of the cytoplasm and other organelles [2]. There are three defined forms of autophagy: macroautophagy, microautophagy and chaperone-mediated autophagy (CMA). Macroautophagy is the process in which cytoplasmic cargo is delivered to lysosomes through double membrane-bound vesicles known as autophagosomes. Vesicles fuse with the lysosome where enzymic protein degradation occurs. In contrast, microautophagy is the process in which lysosomes take up cytoplasmic components directly through invagination of the lysosomal membrane. CMA is a secondary response to starvation and, unlike the aforementioned processes, directly involves the translocation of unwanted proteins to the lysosome under the control of chaperone proteins such as Hsc-70 [2,3]. In most cases macroautophagy is the primary means by which cytoplasm-to-lysosome delivery occurs. Therefore, macroautophagy is more commonly referred to as ‘autophagy’.

The autophagic program of cell degradation was initially thought to be a response to stimuli such as starvation to allow for recycling and regeneration of cellular macromolecules. However, recent studies have shown that autophagy has a significant role in the maintenance of homeostasis, even in non-starved cells [4]. As such, autophagy has a greater physiological and pathological role in higher eukaryotic cells, including the regulation of cell death, the elimination of microorganisms, the control of intracellular protein and organelle turnover, adaptation to starvation and ageing and tumour suppression [5,6]. Additionally, autophagy modulates innate and adaptive inflammatory responses, antigen presentation, and pathogen clearance [7]. Age-related decreases in autophagic activity have been proposed to contribute to the pathogenesis of ageing diseases [8]. Taken together, autophagy plays a vital role in cell homeostasis, and the dysregulation of autophagy is a feature of numerous diseases.

Human uveal melanoma (UM) arises primarily from melanocytes of the iris (accounts for ~6% of UM cases), ciliary body (~4% of UM cases) and choroid (~90% of UM cases) [9]. The symptoms of UM mainly include blurred or distorted vision and visual field loss or photopsia. However, patients may also be asymptomatic resulting in delayed diagnosis and treatment. In addition, multiple referrals may be required before a diagnosis, which may also contribute to its delayed diagnosis [10].

UM consists of ~5% of all cases of melanoma and has an incidence that varies across different regions (ranging from ~1 to 9 per million population per year). UM primarily impacts Caucasians, which aligns with its major risk factors of fair skin, light-coloured eyes and certain hereditary conditions such as the BAP1-tumour predisposition syndrome. Additionally, most UM patients are between the age of 50 and 70 years, and the incidence in adolescents is very low (<1%) [11,12,13]. The incidence of UM is increased at higher latitudes [14].

While relatively rare, UM is lethal and has a high mortality rate of ~30% at 5 years and ~45% at 15 years [15]. Moreover, UM has a high metastatic rate, with the liver being the most common target organ. In metastatic UM, the median survival is ~4–5 months with a 1-year survival rate of 10–15% [9].

The high mortality rate of UM is primarily due to a deficiency of information regarding its pathogenesis and the absence of effective treatments [16]. Current approaches to treat primary UM mainly include enucleation and conservative treatments such as brachytherapy and radiotherapy. However, in many cases, tumours may have already metastasised or advanced locally to the point where conservative treatment is no longer effective. At present, validated methods to diagnose the early onset of disease are not available [17,18,19].

Current drug research has brought new options into light, particularly tebentafusp. Tebentafusp is an FDA approved treatment of HLA-A*02:01 positive unresectable or metastatic UM. It induces immunoreactions through T cell activation [20]. However, it is only effective for the specific genotype carried by less than 50% of the Caucasian population limiting its application in the overall UM patient cohort [21].

Melphalan is also recently approved for UM patients with unresectable hepatic metastases affecting less than 50% of the liver. The randomized controlled multicentre phase III Trial deemed that it has the capability of increasing the hepatic progression free survival from 49 days to 245 days [22]. Nonetheless, ~90% of the patients experienced adverse effects during 72-h post perfusion with thrombocytopenia (74%) and anaemia (60%) being the most common adverse events. Moreover, 91% of patients experienced toxicities between 72 h to 30 days after dosing or until the start of the next cycle of treatment with majority cases of bone marrow suppression. Death was also seen in this trial with two patient deaths associated with bone marrow suppression and one associated with progressive hepatic failure with near-complete hepatic replacement [22]. Similar side effects have also been reported in the SCANDIUM Trial that resulted in one patient death due to multiorgan failure from liver dissection causing liver necrosis and aspiration pneumonia [23]. Albeit that liver is the most common site for UM metastasis, it only envelopes around 60% of patients [24]. This limits those who are eligible for treatment and the associated adverse events are often severe with dire consequences. Thus, there remains an urgent need to develop new prognostic methods and therapeutic approaches in UM. Literature indicates that autophagy is important for developing biomarkers and identifying therapeutic targets in UM. Thus, this review summarizes the current information regarding autophagy in UM that may provide insights in its diagnosis and treatment.

### 1.1. Selective and Non-Selective Autophagy

Due to differences in the stimuli for initiation, autophagy is often classified into selective and non-selective autophagy. Non-selective autophagy is essentially a sequestration and bulk degradation system in which cells turn over and intracellular components are recycled [5,25]. Non-selective autophagy is activated in response to starvation to maintain the cellular supply of lipids, amino acids, carbohydrates, and nucleotides. On the other hand, selective autophagy is activated when there is a need to remove or recycle harmful or unwanted cellular components. Thus, autophagy maintains the quality control of organelles and cellular structures, including cytoplasmic aggregates, endoplasmic reticulum, exogenous proteins, lipid droplets, mitochondria, peroxisomes, and ribosomes [26].

Although different signals activate the alternate forms of autophagy, selective and non-selective autophagy utilize common machinery [27]. The major distinction between non-selective and selective autophagy is the ability to distinguish functional and non-functional organelles or misfolded and correctly folded proteins. The criteria that enable these distinctions to be identified in cells remain largely unknown. However, it has been hypothesised that the cargo itself provides the template that determines the dimensions of the phagophore through specific membrane protein recognition [26,27,28]. This is the “cargo-ligand-receptor-scaffold” model. The interaction between the receptor and scaffold controls cargo recruitment to the phagophore assembly site, where an autophagosome forms. The cargo-ligand-receptor-scaffold model allows selective autophagy to meet at least three essential criteria that differ from non-selective autophagy: (1) the cargo must be specifically recognized; (2) the cargo must be efficiently transported to a new autophagosome; and (3) non-specific material must be excluded from the autophagosome [29]. In selective autophagy, the autophagosomes are smaller and are more tightly bound to the cargos.

In yeast, autophagy-related protein (ATG)-11 is the most common scaffold protein that is involved in several types of selective autophagy, e.g., the cytoplasm-to-vacuole targeting pathway, mitophagy (the removal of damaged mitochondria), and pexophagy (the turnover of peroxisomes). However, a functional counterpart of ATG11 is yet to be discovered in mammals. Nevertheless, it is known that in both yeast and mammals, the receptor proteins subsequently bind to ATG8, which is one of the LC3 family proteins, through either the AIM (Atg8-family-interacting motif) or LIR (LC3-interacting region) sequence. AIM and LIR allow for direct binding of cargo to the autophagy machinery [30].

### 1.2. Autophagy Pathway

**Figure 1:**
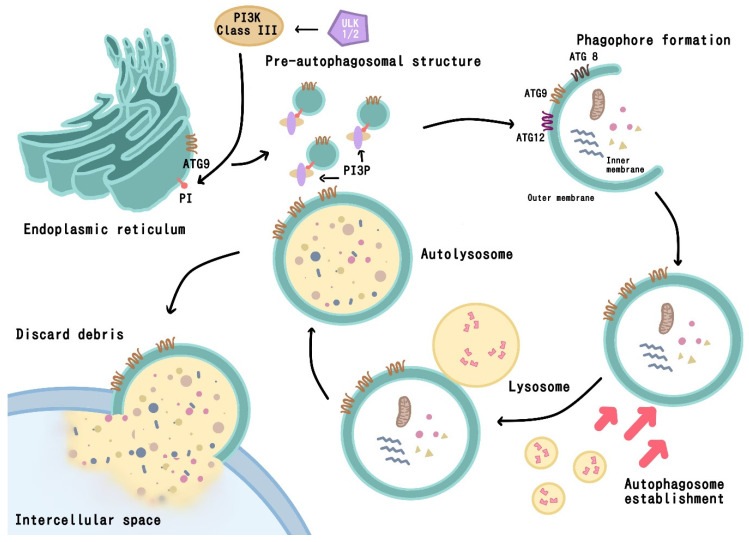
Overview of the autophagy pathway.

The autophagy pathway can be separated into three main stages: the formation of the phagophore, autophagosome establishment and autolysosome formation/material degradation (Figure 1) [27]. During the first stage, an isolation membrane (or phagophore) is formed using lipids from the endoplasmic reticulum (ER), Golgi apparatus or endosome. The phagophore then elongates and engulfs intracellular components such as protein aggregates, organelles, and ribosomes. Membranes close and are then fused with lysosomes to form autolysosomes contain the enzymes that degrade autophagic cargos [31].

Autophagy commences with the activation of Unc-51-like kinase 1 (ULK1) and -2 (ULK2) and the formation of complexes with ATG proteins in mammalian cells. For example, ATG9 mediates elongation of the initial sequestering compartment and phagophore formation. The class III PI 3-kinase complex I (PI3KC3-C1) is then activated by ULK1 and phosphorylates phosphatidylinositol (PI) on the endosomal membrane to generate PI-3-monophosphate (PI3P), a key membrane marker for intracellular trafficking and autophagosome formation. Two other ATG proteins—ATG8 and ATG12—are the drivers of autophagosome formation [26,27,32,33,34]. This process is modulated by the AMP kinase/mammalian target of rapamycin (AMPK/mTOR) pathway that regulates ULK1 [27,34]. Disruption of the autophagy pathway may lead to the development and progression of human diseases, particularly cancers and neurodegenerative diseases [35].

Unc-51-like kinase 1 (ULK1) and -2 (ULK2) activate autophagy by binding with ATG9, to form pre-autophagosomal structures that separate from the endoplasmic reticulum (shown here), Golgi apparatus, mitochondrion, or recycled endosomes. Pre-autophagosomal structures then assemble to form phagophores that activate ATG12. Lysosomes fuse with an autophagosome to produce an autolysosome so that the enclosed cargo may be lysed and then either recycled or removed.

## 2. Autophagy in Human Diseases

As a key mechanism in the maintenance of homeostasis, autophagy regulates cellular energy production and macromolecular synthesis through pro-survival pathways. Autophagy is dysregulated in human diseases, including cancer (e.g., prostate, breast, ovarian) and neurodegenerative disease (e.g., Alzheimer’s and Parkinson’s disease) [36]. The evidence that autophagy participates in numerous disease processes underscores its importance as a potential target for disease treatment.

### Autophagy in Cancers

The role of autophagy in cancer is somewhat controversial. Some studies suggest that autophagy is a pro-tumorigenic mechanism since it enables metabolite recycling and supports tumour metabolism, which increases cancer cell survival. Other studies suggest that autophagy acts as a tumour suppressor that prevents cancer initiation by facilitating the elimination of misfolded or aggregated proteins and damaged organelles [37].

In immortalised mouse kidney cell lines, autophagy is activated in response to long-term starvation, which decreases cell size. This suggests that the clearance of cytosolic proteins and organelles may aid survival under conditions where apoptosis is inhibited [38]. Since defects in apoptosis can promote tumorigenesis, the activation of autophagy is required for the removal of defective intracellular molecules that may otherwise promote tumorigenesis [39]. The inhibition of autophagy leads to the accumulation of unnecessary cytosolic materials and promotes metabolic stress [40,41]. The BECN1 gene encodes the major autophagic regulator beclin-1 (also known as ATG6), and defective beclin-1 can inactivate autophagy [42]. Autophagy may be inactivated either directly—via allelic loss or defective BECN1 or ATG6—or indirectly—through constitutive activation of the pro-survival phosphatidylinositol 3-kinase (PI3K) pathway [43,44,45]. Bhutia et al. found that autophagy was attenuated by oncogenic mutations during tumour initiation [46]. Importantly, the BECN1 gene is deleted in around 50% of breast, ovarian and prostate cancer cases [47,48]. Bax-interacting factor-1 (Bif-1) has a role in membrane dynamics and is another important gene that modulates apoptosis and autophagy. Bif-1 production is decreased in gastric and prostate cancer and Bif-1 null mice show increased susceptibility to tumorigenesis [49]. These findings are consistent with an anti-cancer role for autophagy.

On the other hand, there is also evidence that autophagy is pro-tumorigenic. Since autophagy maintains access to nutrients and removes unwanted cellular material, this can increase the resilience of the tumour cell to metabolic stress, such as hypoxia [39,50]. Furthermore, autophagy allows tumour cells to self-digest during prolonged stress, which prevents cell division and promotes dormancy [39]. Such cells retain the capacity to resume growth and tumorigenic activity once conditions have normalized [37]. In support of this contention, Degenhardt et al. [39] reported that the inhibition of autophagy in cells in which apoptosis is defective, promoted tumour cell death, suggesting that autophagy may disrupt tumour suppression. Finally, deletion of the autophagy regulator FIP200 gave rise to multiple defects in autophagy leading to tumour suppression in the MMTV-PyMT mouse model of breast cancer. Gene expression profile of the tumours in mice determined that loss of FIP200 had no effect on apoptosis within the tumour site but increased immune responses through genome-wide alteration of genes encoding proteins involved in response to Type I interferons (IFN) stimulation and other immune responses such as immune cell infiltration and cytokine production and facilitated the removal of malignant cells [51].

Although it remains unclear why autophagy exhibits different actions at different stages of cancers, this information may have prognostic value. For example, different autophagy-related genes (ARGs) have recently been found to correlate with patient survival profile and may be further developed as tumour biomarkers in breast, glioblastoma, and colon cancer [52]. The role of calcium in tumour prognostics has also been explored with a focus on autophagy. As BAP1 along with other tumour suppressors regulate calcium release from the ER to drive cell death. Truncating mutations in the tumour suppressor gene BAP1 increased susceptibility to developing malignant pleural mesothelioma (MM). Findings show BAP1 mutations resulted in downregulation of calcium dynamics and as autophagy is regulated by these cellular processes, it is hypothesised BAP1and autophagy may be a regulator of MM development [53,54]. Mutations within oncogenes such as the splicing-factor-3B-subunit-1 (SF3B1) has been studied as a novel treatment target associated with autophagy. Fuentes-Fayos et al. demonstrated inhibition of SF3B1 bring about significant alterations within the AKT-mTOR and ß-catenin signalling pathways which is closely associated with glioblastoma (GBM) progression and initiation while also having direct involvements with autophagy regulations suggestive of changes in SF3B1 can influence GBM through autophagic pathways [55,56]. Autophagic processes has also been widely explored in other melanomas such as cutaneous melanoma (CM). Being the most common type of melanoma, connection between autophagy and CM has been comprehensively examined. Although the precise function and effect of autophagy in CM remains controversial with reports of it being both tumorigenic and tumour suppressive [57], further studies have indicated one of the most common mutations occurring in CM—the BRAF^V600E^ mutation is closely related to inhibition of autophagic cell death through transcription factor EB (TFEB). Li et al. demonstrated BRAF inhibitor-induced autophagy through activation of TFEB via ERK signalling pathway inhibition [58]. Furthermore, a key autophagy regulator, AMPK, is inhibited by BRAF^V600E^ activity hereby promoting melanoma cells proliferation [59]. Herewith, demonstrating the diversity of autophagy in different cancers.

Autophagic mediators are potential targets for cancer prognosis and the development of new therapeutics in common cancers, but little is known about their role in rare cancers. Human UM is a rare cancer and several studies have explored the potential of ARGs as prognostic biomarkers in UM.

## 3. Autophagy in Uveal Melanoma

### 3.1. Current Treatments Affecting Autophagy in UM

Autophagy has been extensively studied in various cancers. However, being a rare cancer, the concept of autophagy largely remains unexplored in UM. UM is characterised by genetic mutations to the paralogous guanine nucleotide-binding protein Gq subunits alpha and alpha-11 (GNAQ and GNA11, respectively) which are observed in 80–90% of tumours [60,61]. However, despite the high incidence of GNAQ/GNA11 mutations, overall UM has a relatively low mutational burden so that targeted treatments based on genetic driver mutations have been difficult to identify [62]. Recent studies have suggested that GNAQ/GNA11 and autophagic pathways may be linked. Ambrosini et al. demonstrated that signalling by mutant GNAQ/GNA11 was impaired by the MEK inhibitor selumetinib and the AKT inhibitor MK2206. AKT and MEK are different signalling pathways that converge at GNAQ/GNA11. Therefore, when inhibitors of these two pathways are used together, there is a synergistic increase in autophagic cell death through activation of AMPK [63]. Furthermore, the combination inhibited tumour growth in xenograft mouse models [63]. These effects were genotype dependent since the autophagic markers beclin1 and LC3 were induced in GNAQ-mutant cells, whereas apoptotic cell death was activated in BRAF-mutant cells, and cells without either mutation underwent cell-cycle arrest [63]. Similar findings were noted with the MAPK inhibitor trametinib in combination with the autophagy and lysosomal inhibitor, chloroquine [64]. These apparent links between GNAQ/GNA11-driver mutations and autophagy now warrant further research to identify potential new treatments in UM.

In PDX isolates of UM, neratinib caused the internalization and degradation of GNAQ and GNA11 that was enhanced by the histone deacetylase inhibitor entinostat [65]. Down-regulation of GNAQ and GNA11 required Beclin1 and ATG5 [65]. The combination of neratinib and entinostat engaged multiple pathways to mediate killing, including ROS-dependent activation of the ATM kinase via the AMPK-ULK1-ATG13-Beclin1/ATG5 axis [65]. The knockdown of ATM, AMPK or ULK-1 prevented ATG13 phosphorylation and the degradation of RAS and Galpha subunits [65]. Over-expression of activated mTOR prevented ATG13 phosphorylation and suppressed killing [65]. Thus, neratinib and entinostat down-regulates oncogenic RAS and the oncogenic drivers present in most UM tumours and promotes autophagic cell death [65]. Indeed, targeting dysregulated AMPK-linked cascades may represent a new strategy in UM treatment. Thus, metformin—an adenosine monophosphate-activated kinase (AMPK) activator—inhibited the proliferation and migration of ocular melanoma cells both in vitro and in vivo and attenuated autophagic influx [66]. It would be of potential interest to pursue these observations and assess whether they may be new treatment modalities in UM.

Natural compounds are also under the spotlight as potential treatment options for UM. The compound (−)-4-O-(4-O-β-D-glucopyranosylcaffeoyl) quinic acid (QA) derived from the endophytic fungus *Penicillium* sp.FJ-1 of *Avicennia marina* has been investigated in UM. Treatment with QA demonstrated potent anti-proliferative effect in a concentration dependent manner. Cell autophagy was induced through upregulating mRNA expression of Beclin-1 and down regulation in LC-3, P62and PI3K signalling. This effect was further assessed in in vivo xenograft mouse model, where QA not only decreased tumour volume but also increased pro-apoptotic protein expression causing overall cell death [67].

Nonetheless, the conflicting nature of autophagy on tumour progression, survival, and suppression is also present in UM. The protective effect of autophagy was elucidated in UM through Annexin A2 receptor (AXIIR). Zhang et al. validated the dual effect of AXIIR in UM. Although overexpression of AXIIR through overexpression resulted in overall decrease in cell viability through apoptosis, this effect was reduced with the activation of autophagy. The use of autophagy inhibitor on AXIIR overexpressed cells resulted in a greater apoptotic death suggesting autophagy to resume a cellular protective role [68]. Similarly, novel drug therapy study using the autophagy inhibitor elaiophylin resulted in induced cell death in UM cell lines (i.e., C918, OCM-1A and Mel270) but not healthy retinal ARPE-19 cell line. Treatment with elaiophylin generated oxidative stress and mitochondrial dysfunction while causing the inhibition of mitophagy. The induced oxidative stress caused subsequent accumulation of defective mitochondria and ultimately cell death. The treatment result was further translated into in vivo xenograft mouse models where treatment with elaiophylin caused a reduction in tumour size as well as an increase in apoptosis [69]. Previous studies have linked autophagy with the resistance of tumours to chemotherapies [70]. The combination of selamectin and cisplatin showed a synergistic effect in inhibiting UM cell growth and in tumour-bearing nude mice [70]. Selamectin inhibited the expression of ATG9B, thus decreasing autophagy [70]. The cisplatin resistance-associated genes PDGFRB, DUSP1, MAST1 and IL11 were also downregulated in UM cells treated with selamectin [70]. These findings provide counter arguments for autophagy in UM. Admittingly, autophagy in UM remains disputed. Thus, new definitive markers for diagnosis and treatment are required to improve patient survival. This review examined the novel findings for UM biomarkers.

### 3.2. Protein Based UM Autophagy Biomarkers

There is an urgent need for new prognostic approaches in UM for potential diagnostic and prognostic tools that also serve as treatment markers. Protein mutations in Table 1 are apparent in many cancers including UM and can serve as both diagnostic tool as well as target for novel drug therapies. Ealy intervention could prohibit the development of metastatic disease and improve survival rates.

A promising autophagy-related biomarker is Beclin-1, which is encoded by the BECN1 gene on chromosome 17q21 (Table 1). BECN1 is essential for autophagosome formation since it facilitates the recruitment of other ATG proteins. Deletion of BECN1 has been reported in human breast, ovarian and prostatic cancer cell lines and BECN1+/− mutant mice exhibit a high incidence of spontaneous tumours, which implies a tumour suppressor function for autophagy [71]. In UM BECN expression is associated with a lower risk of metastasis and an increase in disease-free survival [71]. Giatromanolaki et al. explored the potential role of BECN1 as a prognostic marker in cohort of 99 UM tumours following enucleation. Survival analysis showed that both under- and over-expression of BECN1 was associated with metastasis and poor disease survival. However, under-expression of BECN1 was related to a slower rate of initial metastasis rate than with overexpression of BECN1 [72]. At present the role of beclin-1 in cancer is not completely clear but it remains a potential biomarker in UM.

The BCL2 19 kD protein-interacting protein 3 (BNIP3) has been assessed as a potential prognostic biomarker in UM (Table 1). BNIP3 is a BH3 containing protein from the BCL-1 family that modulates cell death, autophagy and cytoprotection. BNIP3 possess the BH3 domain (Bcl-2 homology) common to both pro- and anti-apoptotic bcl-2 family proteins. However, unlike other Bcl-2 proteins, BNIP3 interacts directly with other BCL-2 family members via its C-terminal transmembrane domain rather than BH3, which underlies its dual effect on cell survival [73]. Apart from activation of apoptotic pathways involved with Bcl-2, upregulation of BNIP3 also induces mitochondrial depolarisation and autophagy [74]. Studies in breast cancer and malignant glioma have reported that increased BNIP3 decreased metastasis and improved treatment outcomes [75,76]. However, conflicting conclusions have also noticed in salivary adenoid cystic carcinoma and non-small cell lung cancer [77,78]. In these tumours, increased BNIP3 led to poorer prognosis and decreased metastatic free survival. Similar findings were made in the cohort study of Jiang et al., which found that the high expression of BNIP3 was associated with hyper-pigmentation, deeper scleral invasion and a poor prognosis in UM [79]. BNIP3 detection could help stratify high-risk patients and identify new therapies targeting BNIP3 as a promising approach to treat UM [79].

Autophagy is also modulated by the PI3K/AKT/mTOR, p53, MAPK and NFκB signalling pathways (Table 1) [80,81,82]. AMPK and mTOR are involved in the autophagic process as the activator and inhibitor, respectively. Notably, mTOR is the key integrator of nutrient signalling and cellular growth factor. It is responsible for autophagy inhibition under nutrient sufficient conditions via preventing the activation of Ulk1 [83]. Protein tyrosine kinase 6 (PTK6) is an mTOR regulator and promotes breast, colorectal and lung tumorigenesis by activating multiple signalling pathways [84,85,86]. Although PTK6 has been explored in other cancers, its involvement in UM is yet to be fully elucidated. However, Liu et al. reported that increased PTK6 expression in UM cells was associated with a poor prognosis [87]. Increased PTK6 activates mTOR and in turn, increases tumorigenesis by promoting the proliferation, migration, and invasion of UM cells and inhibiting autophagy [87]. PTK6 binds to SOCS3 in UM cells, so that targeting the SOCS3-PTK6 signalling axis might be a novel and promising therapeutic strategy for patients with UM [87]. The trial of neratinib and entinostat also demonstrated the therapeutic role of mTOR as well as other crucial signallings such as AMPK-ULK1-ATG13-Beclin1/ATG5 axis, which study indicated that the regulation of mTOR activity and its control of autophagosomes played a key role in treatment response to neratinib/entinostat combination [65]. The drug combination increases the phosphorylation of ULK-1 S317 and reduces phosphorylation of mTOR. Furthermore, this study demonstrated that the activation of mutant form of mTOR supressed the drug combination-induced activation of ATM, AMPK and ULK-1 resulting in decreased killing effect, which suggested that mTOR activation plays a vital role in cellular autophagic processes [65].

Mutations in the BRAF gene are common in cutaneous melanoma but not in UM [88]. However, from gene profiling studies, this mutation was detected in some cell lines [89,90,91]. Indeed, in BRAF V600E mutant UM cells vemurafenib produced cell death by inhibiting BRAF and mTOR [92]. Thus, the mTOR-linked signalling pathway is implicated in UM survival.

**Table 1:** Summary of key genes/proteins as potential late biomarkers for autophagy.

Gene/Protein	Physiological Role(s)	Clinical Advantage/Disadvantages	References
BECN1/Beclin-1	- Important in formation of autophagosomes	- A key component of autophagy, a promising prognostic factor - Inconclusive role in tumorigenesis	[71,72]
BCL2 19 kD protein-interacting protein 3 (BNIP3)	- A BCL-1 family member - Modulates cell death, mitochondrial depolarisation and autophagy	- may enhance tumour killing effect - Hard to be therapeutic target as many Bcl-1 family proteins with similar functions	[73,74,75,77,79]
Mammalian target of rapamycin (mTOR)	- Important for cell survival and cell death	- Well studied with detailed and readily available pathways and pharmacotherapy - May have off-target effects due to versatility	[80,81,82,87,88,92]

### 3.3. Gene Based UM Biomarkers

With advancements in technology, patient-based tumour gene sequencing and related therapies become predominant. Early identification of genetic mutations and variations can serve as prognostic and treatment tools to aid in patient recovery and minimise metastasis.

ARGs have been evaluated increasingly as potential cancer biomarkers [39,41,42,50]. In recent years, several ATG genes have been identified as potential biomarkers for use in cancers such as breast cancer, colon cancer and glioblastoma [93,94,95]. ATG genes and other ARGs could also be developed as UM biomarkers. Recently, Zheng et al. identified a robust 9-ARG signature that was prognostic of survival in a cohort of 230 patients with UM [96]. The Cancer Genome Atlas (TCGA) UM cohort was used as a training set to identify the signature that was then validated using four other cohorts of 150 UM patients [96]. The 9-ARG signature was distinctively enriched in high-risk UM patients and was associated with several cancer hallmarks, including angiogenesis, IL6-JAK-STAT3 signalling, reactive oxygen species production and oxidative phosphorylation, as well as immune-related functional pathways and immune cell infiltration [96]. Moreover, the ARG signature seemed to distinguish between low- and high-risk UMs [96]. Although the small sample size in the investigation highlights the need for caution, ARG analysis could well be a promising new approach for further evaluation in UM.

Autophagy-associated long non-coding RNA (lncRNA) may also be potential prognostic indicators in UM (Table 2). lncRNA are RNAs of 200 nucleotides or longer that do not encode protein and that were previously considered to be transcriptional noise [97]. However, lncRNA may be important in cellular development, including epigenetics, chromatin remodelling and genetic imprinting [98,99]. Dysregulation of lncRNA is associated with tumorigenesis [100,101,102]. Multiple lncRNA are linked to epigenetic and genomic modifications, and are both tumour suppressors and oncogenes [103]. Detailed analyses, such as that performed by Li et al., have demonstrated that induction of the lncRNA ZNNT1 by rapamycin upregulated ATG12 expression in UM cells and inhibited tumorigenesis by activating autophagic cell death [104]. Specific lncRNA might be developed as biomarkers and treatment targets of UM [105,106]. Another novel discovery of LnRNA is LINC01278. This gene is closely related to autophagic genes in UM and acts as a double-edged sword where it can promote tumour metastasis. On the other hand, LINC01278 inhibited the progression cancer progression through MiRNA activation. Liu et al. discovered the inhibitory effects of LINC01278 on UM. resulting data proposed LINC01278 to have inhibitory effects on UM where overexpression caused a decrease in proliferation, migration, and invasion levels of UM cell line OCM-1 and MUM-2B. This was carried out through activating the autophagic pathway with P62 up regulation and decreasing LC3 II/LC3 ratio along with mTOR signalling pathway. This finding was also translated onto xenograft mouse models where upregulation of LINC01278 reduced the volume and weight of tumour compared to control. With these as the basis, more research into the insight of the function and use of LnRNA is encouraged to uncover potential diagnostics and treatment options for UM [107].

As with the lncRNA, micro-RNA (miRNA) may be potential UM biomarkers and anti-cancer drug targets (Table 2). miRNAs are single non-coding strands of 21–23 nucleotides that modulate gene expression by regulating mRNA decay or translation inhibition/activation [108]. An individual miRNA can target multiple genes—a property that may be advantageous in the development of new anti-cancer therapies [109]. Targeting miRNA has been evaluated in gastric cancer, hepatocellular carcinoma, lung cancer, and oesophageal cancer [110,111,112,113]. Multiple miRNAs appear to be dysregulated in UM [114,115,116]. Certain miRNAs are potential prognostic markers for UM and clustering tumours according to miRNA expression appears to correlate with metastatic risk [117,118]. Wu et al. found using cell and mouse models that overexpression of miRNA miR-608 down-regulated the tumour promoting gene HOXC4, that was attenuated by overexpression of metastasis-associated lung adenocarcinoma transcript 1 (MALAT1), which is implicated in cancer as a pivotal regulator of pro-tumorigenic signalling [119]. Knockdown of HOXC4 suppressed UM cell migration, proliferation, invasion, and cell cycle progression (81). Targeting MALAT1 may be a viable approach in the development of new UM treatments. MALAT1 was upregulated in UM tissues and its knockdown has been found to suppress UM cell proliferation, colony information, invasion, and migration [120].

Drug treatments that modulate miRNA expression have also been explored to some extent and exhibit promising activity. Sun et al. evaluated the flavonoid genistein in UM. Inhibition of UM cell growth by genistein was time- and dose-related [121]. In in vivo studies genistein inhibited xenograft growth [121]. Genistein modulated miR-27a expression and, in turn, the Zinc Finger And BTB Domain Containing 10 gene that regulates RNA polymerase related DNA transcription [121]. Further study is now warranted to evaluate other miRNAs in UM progression, and whether they may be targeted for therapeutic purposes.

Genetic profiling with the ever-expanding patient database is becoming the frontier of cancer research. Correlation between genetic mutations and patient prognosis has been utilised in many cancers, but this area of study is still generally lacking in UM (Table 2). With increased UM patient data, this field of research will provide imperative information to the diagnostics and treatment paradigm of UM. By using the TCGA-UVM gene database then validating it with gene expression database (GSE84976, GSE 22138) Liu et al. was able to successfully correlate autophagy and immune related genes to reveal four novel genes (PRKCD, MPL, EREG, and JAG2) in UM that has been correlated with prognosis in other malignancies [122]. The risk scores generated for the four genes are closely related to chromosome 3 status and was an accurate interpretation of patient prognosis when used in the three patient databases. Further correlation identified immune changes such as fraction of CD4, CD8, regulatory T cell and NK cell activation, monocytes, macrophages M1, and mast cells in the high-risk score group to significantly differ from UM patients in the low-risk score group. Additionally, association between drug resistance of two common drug among the three database and risk score determined significant differences in drug sensitivity where high risk group could be more sensitive to chemotherapy [122].

In the recent report, Jin et al. applied multi-omics approaches to analyse UM patients’ clinical and molecular features, which study discovered six novel prognostic biomarkers in relation to autophagy in UM (i.e., SPHK1, HTR2B, FEZ1, EEF1A2, HAP1, and GRID1) [123]. The high expression of these genes has been found to be associated with poor patient prognosis and tumour progression. Thus, experimental evidence is highly desired to validate the clinical relevance of these newly identified marker genes in UM.

Overall, multiple potential prognostic biomarkers that regulate autophagy in UM have been found. Greater understanding of the molecular roles of autophagy and its regulation in UM could clarify whether these proteins may be useful in understanding and diagnosis of UM progression and whether they may be potential drug targets.

**Table 2:** Summary of key genes/RNAs as potential biomarkers for autophagy.

Gene/RNA	Physiological Role(s)	Clinical Advantages/Disadvantages	References
Autophagy related genes (ARGs)	- Set of genes required for autophagy. - Mutations of ARGs may result in issues with the autophagy process.	- Multiple targetable genes allow for combination therapy. - Ubiquitous, difficult to differentiate tumour from healthy cells.	[39,41,42,50,96,123]
Long non-coding RNA	- Non protein coding RNA. - Effect on cellular regulatory functions.	- Multiple targetable genes allow for combination therapy. - Mutations in the genes allow for targeted therapy. - Involved in cellular development, such as epigenetics, chromatin remodelling and genetic imprinting.	[97,98,99,103,105,106]
Micro RNA (miRNA)	- Highly conserved non-coding RNA molecules. - Involved in the regulation of gene expression.	- Multiple targetable genes may allow for combination therapy. - Difficult to be considered as a target for pharmacotherapies due to its versatility in the body.	[108,109,111,114,117,118]
Genetic profiling	- Mutation identification through high output methods. - Genetic mutation can result in cancer formation.	- Can pinpoint UM based mutation. On different cell lines - Can easily verify findings on different databases. - May not translate directly into clinical situation.	[122]

## 4. Conclusions

Autophagy is a key process in cellular homeostasis and its activation promotes the degradation and recycling of damaged cells, abnormal cellular materials, and removal of toxic materials. The activation of autophagy is implicated in several cancers, including UM. Although its function in UM remains largely unclear, autophagy mechanisms and mediators have emerged as promising therapeutic targets that could be used to develop new treatment options for UM where there are no effective drugs at present. As key autophagy-related genes, RNAs and proteins may have distinct roles in tumour and non-tumour cells (e.g., mutations impacting on function), targeting these genes/proteins/RNAs may be a viable way to develop therapeutics for tumour cells. In addition, identifying novel biomarkers based on autophagic mediators may be used to prolong patient survival where currently the prognosis is bleak.

## Abbreviations

AIM: Atg8-interacting motif; AKT: Protein kinase B; ARGs: autophagy-related genes; ATG: Autophagy related; Bcl-2: B-cell lymphoma 2; BECN1: Beclin 1 gene; BH3: BCL-2 homology 3; BNIP3: BCL2 Interacting Protein 3; BRAF: proto-oncogene B-Raf; CMA: Chaperone-mediated autophagy; ER: Endoplasmic reticulum; FIP200: Focal adhesion kinase family interacting protein of 200 kD; GNA11: G Protein Subunit Alpha 11; GNAQ: Guanine nucleotide- binding protein G(q) subunit alpha; HOXC4: Homeobox C4; Hsc-70: Heat shock cognate 71 kDa protein; IFN: Type I interferons; LAMP-2A: Lysosome-associated membrane protein type 2a; LC3: Microtubule-associated protein 1A/1B-light chain 3; LIR: LC3-interacting region; lncRNA: Long non-coding RNA; MALAT1: Metastasis Associated Lung Adenocarcinoma Transcript 1; MAPK: Mitogen-activated protein kinases; MEK: Mitogen-activated extracellular signal-regulated kinase; mHTT: Mutant Huntingtin protein; miRNA: MicroRNA; mTOR: Mammalian target of rapamycin; NFκB: Nuclear factor kappa-light-chain-enhancer of activated B cells; p53: tumour protein p53; PI: Phosphatidylinositol; PI3K: Phosphoinositide 3-kinase; UM: uveal melanoma.
